# P-1031. Frequency and resistance patterns of Candida auris causing candidemia in 3 teaching hospitals in Illinois: a routine data analysis

**DOI:** 10.1093/ofid/ofae631.1221

**Published:** 2025-01-29

**Authors:** Jose Luis Paredes, Erik LaChance, Peguy Saad

**Affiliations:** Advocate Health Illinois Masonic Medical Center, Chicago, IL, USA., Chicago, Illinois; Advocate Health Illinois Masonic Medical Center, Chicago, IL, USA., Chicago, Illinois; Advocate Health Illinois Masonic Medical Center, Chicago, IL, USA., Chicago, Illinois

## Abstract

**Background:**

Despite a reported increase in the prevalence of *Candida auris* in the US, a potential emergent multidrug-resistant health care associated pathogen, data on its prevalence and drug resistance patterns is still scarce.

**Methods:**

Sub-analysis or routinely collected data from 3 teaching hospitals in Chicago, Illinois. Isolates from patients with positive blood cultures for any species of Candida between April 2022 and April 2024 were included. The percentage of samples positive for *Candida auris*, considering one isolate per patient, and the prevalence or resistance to several antifungals (echinocandins, azoles, and amphotericin B) was reported.

**Results:**

Among 422 candidemia isolates, 210 (49.8%) were *Candida auris*, isolated from 64 patients: 23 (36.5%), 34 (54.0%) and 6 (9.5%) from 2022, 2023 and 2024 respectively. Four isolates (6%) were resistant to echinocandins, nine (14%) isolates were resistant to fluconazole and one (2%) was resistant to amphotericin B.

**Conclusion:**

A large proportion of candidemia isolates were caused by *Candida auris*. An important percentage was resistant to fluconazole and to echinocandins, supporting amphotericin B or high dose echinocandins as first line therapy.

**Disclosures:**

**All Authors**: No reported disclosures

